# Universal properties of non-Hermitian viscoelastic channel flows

**DOI:** 10.1038/s41598-023-27918-4

**Published:** 2023-01-19

**Authors:** Yuke Li, Victor Steinberg

**Affiliations:** Department of Physics of Complex Systems, Weizmann Institute of Science, 7610001 Rehovot, Israel

**Keywords:** Fluid dynamics, Mechanical engineering

## Abstract

An addition of long-chain, flexible polymers strongly affects laminar and turbulent Newtonian flows. In laminar inertia-less viscoelastic channel flow, the supercritical elastic instability of non-normal eigenmodes of non-Hermitian equations at finite-size perturbations leads to chaotic flow. Then three chaotic flow regimes: transition, elastic turbulence (ET), and drag reduction (DR), accompanied by elastic waves, are observed and characterized. Here we show that independently of external perturbation strength and structure, chaotic flows above the instability onset in transition, ET, and DR flow regimes reveal similar scaling of flow properties, universal scaling of elastic wave speed with Weissenberg number, *Wi*, defined the degree of polymer stretching, and the coherent structure of velocity fluctuations, self-organized into cycling self-sustained process, synchronized by elastic waves. These properties persist over the entire channel length above the instability threshold. It means that only an absolute instability exists in inertia-less viscoelastic channel flow, whereas a convective instability, is absent. This unexpected discovery is in sharp contrast with Newtonian flows, where both convective and absolute instabilities are always present in open flows. It occurs due to differences in nonlinear terms in an elastic stress equation, where except for the advective term, two key terms describing polymer stretching along the channel length are present.

## Introduction

Inertialess viscoelastic flows with curvilinear streamlines exhibit normal mode elastic instability and elastic turbulence (ET) at sufficiently large Weissenberg number, *Wi*, and Reynolds number, $Re\ll 1$^1–3^. The flow becomes unstable due to the elastic stress field generated by polymers stretched along curved streamlines resulting in a bulk force in the direction of curvature. The latter induces a back-reaction on the flow, causing a linear instability by the fastest, exponentially growing normal mode^1–3^. Here $Wi\equiv U_{mean}\lambda / d$ is the ratio of elastic stress to stress dissipation due to relaxation^4^ and $Re\equiv U_{mean} d \rho /\eta$, where $U_{mean}$ is the mean stream-wise flow velocity measured by the flow discharge, $\lambda$ is the longest polymer relaxation time, $\rho$ and $\eta$ are the solution density and dynamic viscosity, respectively, and *d* is the characteristic vessel size.

Nevertheless, this instability mechanism becomes ineffective in parallel shear flows with zero curvature streamlines, such as pipe, channel, and plane Couette flows at $Re\ll 1$^2,5^, whose linear stability is proved^6,7^. However, linear stability does not imply global stability. Indeed, Newtonian parallel shear flows become unstable to finite-size perturbations at finite *Re*, despite their proven linear stability^8^. To explain such instability, a new mathematical concept of the non-normal (non-orthogonal) eigenmodes is introduced^9,10^. Contrary to normal eigenmodes of a Hermitian operator, the non-normal eigenmodes appear in a non-Hermitian operator^8^. As well known, the linearized Navier-Stokes equation (NSE) (or Orr-Sommerfeld equation) for Newtonian parallel shear flows is non-Hermitian and proved to be linearly stable towards the normal modes in the limit of $Re\rightarrow \infty$, where the Orr-Sommerfeld equation becomes Hermitian. However, at finite *Re*, it is prone to transient algebraic temporal growth of non-normal eigenmodes, whereas linear normal modes decay exponentially since the flow is linearly stable^8–10^.

Theoretical and numerical simulation studies of the non-normal mode instability in viscoelastic channel and plane Couette flows at $Wi\gg 1$ and $Re\ll 1$ (and so $El\gg 1$, where $El=Wi/Re$ is the elasticity number) consider only a linear transient stage of the non-normal mode elastic instability^11–15^. Then during the linear growth of the transient non-normal modes, transient coherent structures (CSs), namely stream-wise streaks and rolls, are generated. Nevertheless, sustained non-normal modes result from nonlinear interactions and cannot be anticipated by such an approach^12,16^.

First experiments in viscoelastic parallel shear flow, conducted in pipes^17^ and later on in straight square micro-channels with strong prearranged perturbations at the inlet^18–20^, report large velocity fluctuations above an elastic instability at critical Weissenberg number, $Wi_c$, contrary to the proved linear stability^6,7^.

Recently, we have published results of experiments performed in a viscoelastic planar channel flow with strong prearranged perturbations generated by an array of obstacles at the inlet^21^. Above the supercritical elastic instability, we quantitatively characterize flow friction, rms (root-mean-square) of velocity and pressure fluctuations, and chaotic stream- and span-wise velocity power spectra at $Wi>Wi_c$ in a transition regime and further at higher *Wi*. The dependence of the flow properties above the instability onset on ($Wi-Wi_c$) and, in particular, the chaotic velocity spectra reveal key features of the non-normal mode instability. The span-wise velocity power spectra, besides power-law decays at higher frequencies, also exhibit peaks at lower frequencies, suggesting the emergence of elastic waves on top of a chaotic flow^21^. Furthermore, the CSs, namely stream-wise rolls and streaks, which show up in the linearized model^11,13,14^ and are experimentally observed above the non-modal instability, are self-organized into cycling self-sustained process (SSP) synchronized by elastic wave frequency. CSs are discovered at $Wi>Wi_c$ in three flow regimes: transition, ET, and drag reduction (DR)^21^. However, unlike Newtonian parallel shear flows, where CSs are found over the entire channel length above the absolute instability^22^, in a viscoelastic channel flow with strong perturbations at the inlet, CSs and elastic waves appear only in a limited range of the channel length downstream of the perturbation source. In the rest of the channel downstream up to the outlet, only the normalized rms stream-wise velocity fluctuations, $u_{rms}/U_{mean}\le 4\%$, are found^21,23^, in contrast to Newtonian parallel shear flows, in spite of the fact that both flows are open^8,22,24^. The open flow is the flow where fluid continuously enters and leaves the main experimental domain. Thus, the recent studies of elastic instabilities produce an impression that strong perturbations are unavoidable to cause instability in viscoelastic pipe and channel flows^17–21,23,25^.

Here, we present results that resolve several key problems by conducting the experiment in a viscoelastic planar channel flow with finite-size perturbations generated by the non-smoothed inlet and six small holes at the top plate along the channel, used for pressure measurements. We intend to elucidate the following basic questions. (i) Are the strong prearranged perturbations the necessary condition for the elastic instability, and if not, does a chaotic flow above the instability onset exhibit the same characteristics, including elastic waves and CSs in three flow regimes^21,23^? (ii) Does the elastic wave speed show the same scaling with ($Wi-Wi_c$), as found in Refs.^21,26,27^? (iii) Do these flow features and significant rms stream-wise velocity fluctuations display only in a limited range of the channel length, as found in Ref.^21^, or over the entire channel length? Does only absolute instability without convective one exist in this open flow?

## Results

### Non-normal mode instability, elastic turbulence, and drag reduction

**Figure 1:**
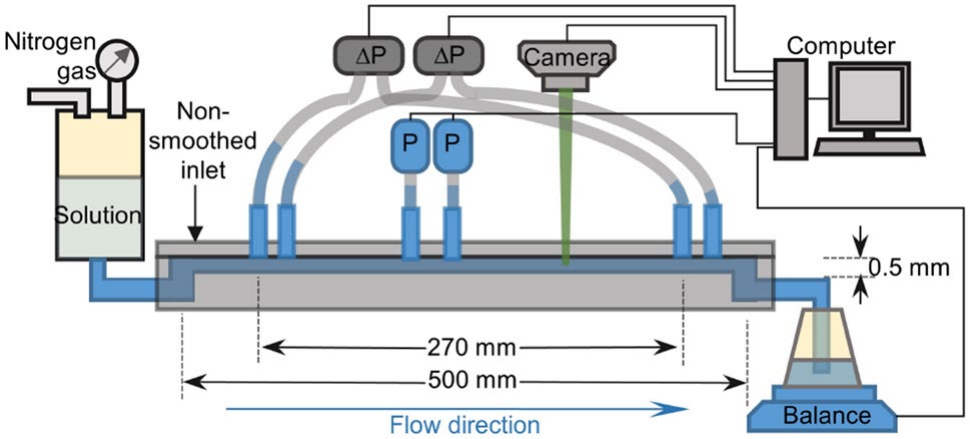
Schematics of the experimental setup. Six holes are used for high-resolution pressure drop and absolute pressure fluctuations measurements in three pressure ranges and in two different positions along the channel. The polymer solution is driven by compressed Nitrogen gas to the straight channel via its inlet. The fluid exiting the channel outlet is weighted instantaneously as a function of time by a computer-interfaced balance. The time-averaged fluid discharge rate is calculated from the weight difference measured by the balance as a function of time. An illuminating sheet for PIV measurement is located at the mid-plane *x*-*z* of the channel.

The experiments are conducted in a straight planar channel of 500(*L*) $\times$ 3.5(*w*) $\times$ 0.5(*h*) mm$^3$ dimensions, shown in Fig. 1. The only possible sources of flow disturbances are the channel’s non-smoothed inlet and six holes in the top plate for pressure drop and absolute pressure fluctuations measurements (see “Methods” section).

**Figure 2:**
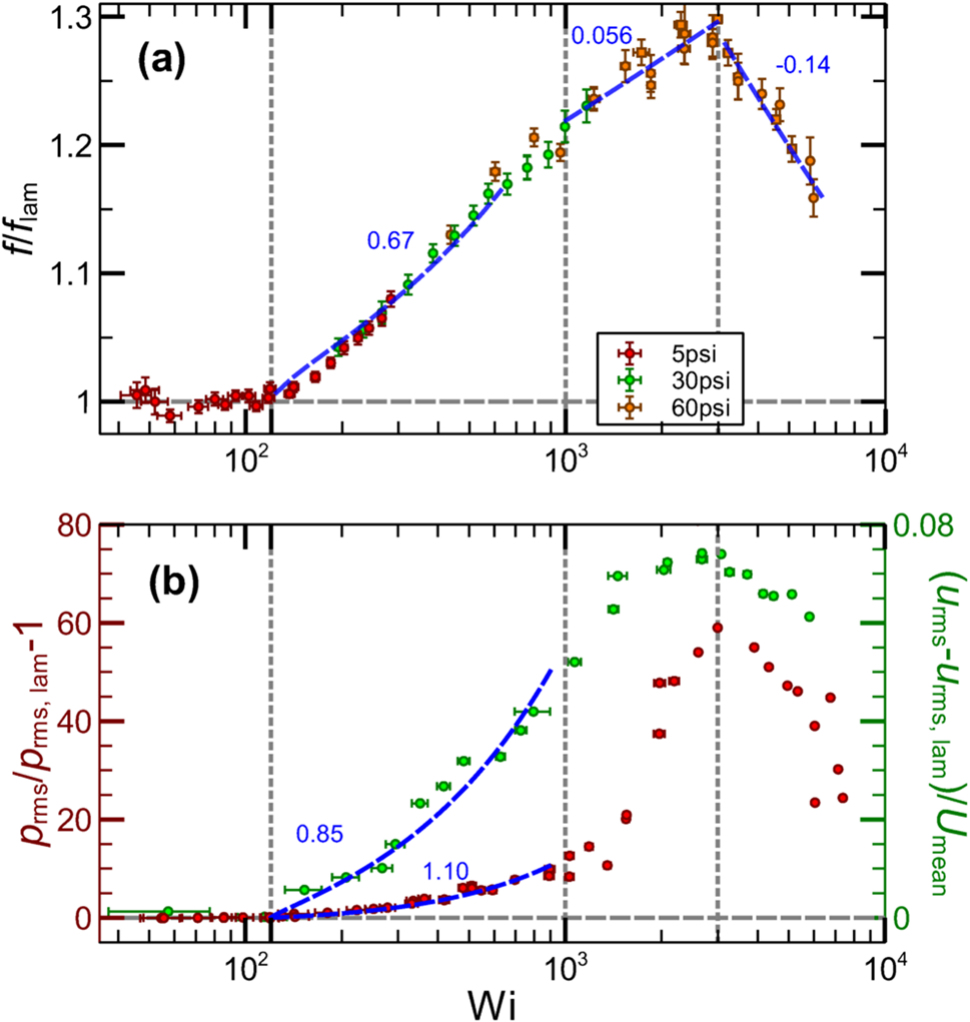
Properties of chaotic flows above the non-modal elastic instability in three regimes. (**a**) The friction factor ($$f/f_{\mathrm {lam}}$$) versus *Wi* in lin-log scales. Two individual pressure drop measurements are conducted at different positions but with the same gap: from *l*/*h*=185 to 725 and from 225 to 765, which are plotted together. Three differential pressure transducers in three ranges are used: 5 (in red), 30 (in green), and 60 (in orange) psi. (**b**) Normalized rms pressure ($$p_{rms}/p_{rms,lam})-1$$ and velocity $$(u_{rms}-u_{rms,lam})/U_{mean}$$ fluctuations versus *Wi* in lin-log scales. The vertical three greys dashed lines separate four flow regimes determined by different scaling of the flow parameters on *Wi*.

In Fig. 2a we present the measurements of the friction factor as a function of *Wi* in high-resolution presentation, $f/f_{\mathrm {lam}}$, calculated from the flow resistance $f=2D_h\Delta P/\rho U_{mean}^2 L_p$ and normalized by the laminar one $f_{lam}\sim Re^{-1}$. Here $U_{mean}$ is the mean stream-wise velocity obtained from the flow discharge measurements (see Methods section for the flow discharge measurements), $D_h=2wh/(w+h)=0.875$ mm is the hydraulic length, $L_p=270$ mm is the distance between two pressure measurement holes and $\Delta P$ is the pressure difference on $L_p$. In Fig. 2b, normalized rms of pressure and velocity fluctuations in different flow regimes are shown.

Four flow regimes are identified by different dependencies of $f/f_{\mathrm {lam}}$ on *Wi* (Fig. 2a). In a laminar flow, $f/f_{\mathrm {lam}}$ equals unity up to $Wi_c=120\pm 10$. Then above $Wi_c$, $f/f_{\mathrm {lam}}-1$ grows in the transition regime with $Wi/Wi_c-1$ as a power-law relation with an exponent $0.67\pm 0.04$. In ET, $f/f_{\mathrm {lam}}$ increases algebraically with *Wi* with the exponent $0.056\pm 0.004$, and decays in DR with the exponent $-0.14\pm 0.01$. These four flow regimes are separated by three greys dashed lines in Fig. 2.

Normalized rms of pressure $p_{\mathrm {rms}}/p_{\mathrm {rms,lam}}-1$ and velocity fluctuations $(u_{\mathrm {rms}}-u_{\mathrm {rms,lam}})/U_{mean}$ versus *Wi* are shown in Fig. 2b. Here *U*(*x*, *y*, *z*, *t*) is the full stream-wise velocity, *U*(*z*) is the stream-wise velocity profile, $u'(x,y,z,t)=U-U(z)$ is the stream-wise velocity fluctuations, and $u_{rms}$ is the rms of stream-wise velocity fluctuations. The velocity fluctuations are measured at the center of the middle plane via PIV. Analogously to the friction factor, both $p_{\mathrm {rms}}/p_{\mathrm {rms,lam}}-1$ and $(u_{\mathrm {rms}}-u_{\mathrm {rms,lam}})/U_{mean}$ versus *Wi* disclose four flow regimes. Above the instability onset, both $p_{\mathrm {rms}}/p_{\mathrm {rms,lam}}-1$ as well as $(u_{\mathrm {rms}}-u_{\mathrm {rms,lam}})/U_{mean}$ as a function of ($Wi/Wi_c-1$) grow algebraically with the exponents $0.85\pm 0.1$ and $1.10\pm 0.1$, respectively. All exponents including the friction factor differ from 0.5 indicating that it is not the normal mode elastic instability^8^.

### Stream- and span-wise velocity power spectra and elastic waves

**Figure 3:**
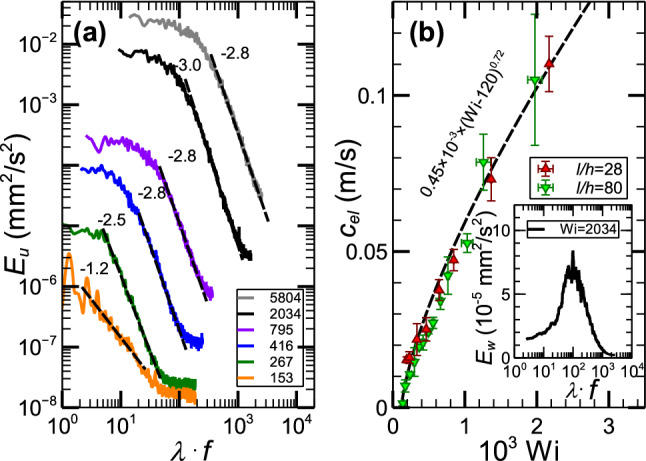
Velocity power spectra and elastic wave velocity as a function of *Wi* in three flow regimes. (**a**) Stream-wise velocity energy spectra, $E_u$, versus frequency normalized by $\lambda$ at *l*/*h*=330 in log-log scales. The velocity for each moment is measured at the channel center. The energy spectra $E_u$ are plotted for different *Wi* in a different color presented in the plot. (**b**) Elastic wave propagation speed $c_{el}$ versus *Wi* at *l*/*h*=28 and 80 in lin-lin scales is fitted by a dashed line with the dependence on $(Wi-Wi_c)$ presented on the plot. The inset shows a peak of elastic wave in the span-wise velocity energy spectrum $E_{w}$ at $Wi=2034$ and *l*/*h*=330 in lin-log scales. The peak value of $E_{w}$ is the intensity of elastic waves that is equal to the square amplitude of the elastic waves.

Figure 3a presents energy spectra ($E_u$) of stream-wise velocity fluctuations versus normalized frequency $\lambda \cdot f$ at *l*/*h*=330, far downstream from the inlet. The exponents of the $E_u$ algebraic decay decrease from − 1.2 to − 2.8 in the transition regime, then further down to − 3.0 in ET, and increase back to − 2.8 in DR. The continuous modes of $E_u$, verifying chaotic flows already above the instability onset in the transition regime, is the strongest evidence of the non-normal mode elastic instability^11,13,14^.

Using the span-wise velocity energy spectra ($E_{w}$) in lin-log scales for different *Wi* (see the inset in Fig. 3b, where the peak in span-wise velocity power spectra is shown), we obtain the dependence of the elastic wave frequency $f_{el}$ on *Wi*, the key indicator of the existence of the elastic waves. The main evidence of the detection of the elastic waves is the dependence of their propagation speed $c_{el}$ on $(Wi-Wi_c)^{\delta }$ to be compared with the early observations^21,23,26,27^. Figure 3b presents $c_{el}$ vs *Wi* in linear scales obtained by measurements of velocity cross-correlation functions at different separations in a stream-wise direction. By fitting the curve with $c_{el}=A\times (Wi-Wi_{c})^{\delta }$, we obtain $A=0.45\pm 0.05\times 10^{-3}$ m/s and $\delta =0.72\pm 0.02$ in a good agreement with our early results found in several flow geometries, namely the flow between two widely separated obstacles hindering the micro-channel flow^26^, and the straight channel flow with strong perturbations due to an array of obstacles at the inlet^21,23^. On the other hand, span-wise propagating elastic waves, discovered in a straight channel with very weak perturbations generated by a small cavity in the top plate in the middle of the channel, are characterized by the same scaling exponent, though about three orders of magnitude smaller coefficient *A* that may be attributed to the strong span-wise confinement resulting in about constant wavelength^27^. Thus, our finding suggests that the stream-wise-propagating elastic wave scaling relation with ($Wi-Wi_c$) is universal.

### Spatial and temporal interface dynamics of stream-wise streaks in three chaotic flow regimes

**Figure 4:**
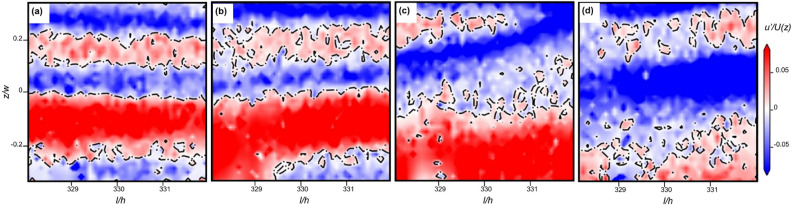
Streak interface dynamics at *l*/*h*=330 and *Wi*=2030 in ET during a single cycle. The images present the instant normalized stream-wise velocity fluctuations, obtained by subtraction from a fully measured stream-wise velocity field and normalized by mean velocity, $u'/U(z)$. The black dashed lines of $u'/U(z)=0$ separate negative (blue) and positive (red) streaks. The images shown at four normalized times $t^*=tf_{el}$ from left to right are 0.20, 0.38, 0.65, and 0.92. The streak interface is gradually disturbed and destroyed resulting in random flow at the cycle end without any secondary instability. Then the next cycle starts.

**Figure 5:**
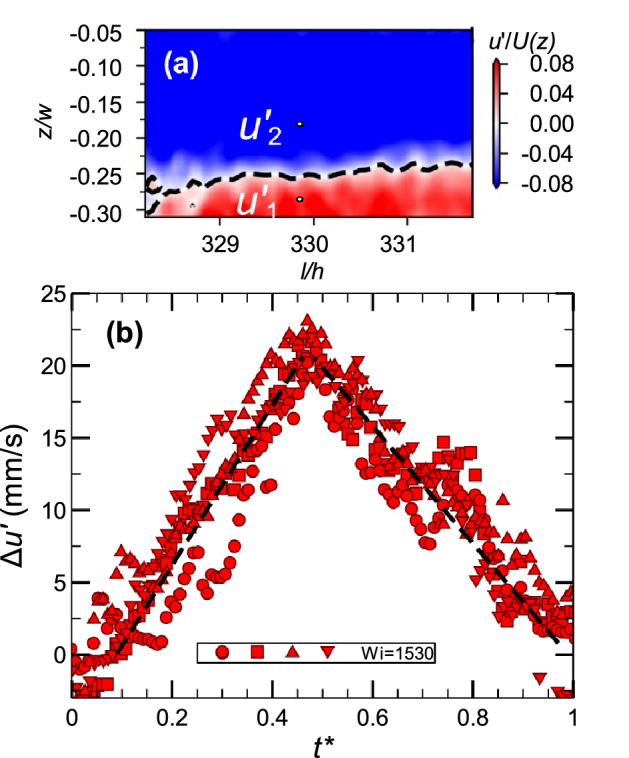
Temporal dynamics of streaks at *l*/*h*=330 at *Wi*=1530 in ET. (**a**) Span-wise-separated two points for calculation of $\Delta u'=u'_1-u'_2$. (**b**) Four runs of $\Delta u'$ for one cycle period normalized by the elastic wave frequency.

To gain better insights into CSs, we broaden the PIV window to the whole channel width and the stream-wise length of $\Delta l/h=4$ to examine the existence of CSs in this channel flow geometry in transition, ET, and DR regimes. To illustrate the structural dynamics in ET, we show in Fig. 4 four consequent images of stream-wise velocity fluctuations in a frame moving with average stream-wise velocity having the span-wise profile in a single cycle synchronized by the elastic wave frequency. The images from left to right show the stream-wise streaks starting with a slightly perturbed interface that further becomes more and more disturbed and finally destroyed, resulting in a random flow toward the cycle end without visible indications of a secondary instability, such as the Kelvin-Helmholtz-like elastic instability reported recently in Ref.^23^. We attribute the absence of the secondary instability to the lower intensity of the elastic waves compared to that found in ET in the planar channel flow with the strong prearranged perturbations at the inlet and much shorter distance from the perturbation source^23^. The stream-wise streaks are found in three flow regimes, though in the transition regime they are observed at $Wi\gtrapprox 350$, where elastic wave intensity becomes sufficiently high. To verify experimentally the periodicity of a cycled SSP with the elastic wave period, we utilize the approach earlier developed at our lab to study the interface dynamics^21,23^ by examining a temporal evolution of the stream-wise velocity fluctuation differences at two specific points across the interface, $\Delta u'=u'_1-u'_2$, as shown in Fig. 5. During each cycle, $\Delta u'$ displays a non-monotonic temporal variation with the same peak value $\Delta u'_{max}$ and cycle period for four cycles in spite of highly fluctuating flows (Fig. 5).

### Spatial range of velocity fluctuations and elastic wave intensity

**Figure 6:**
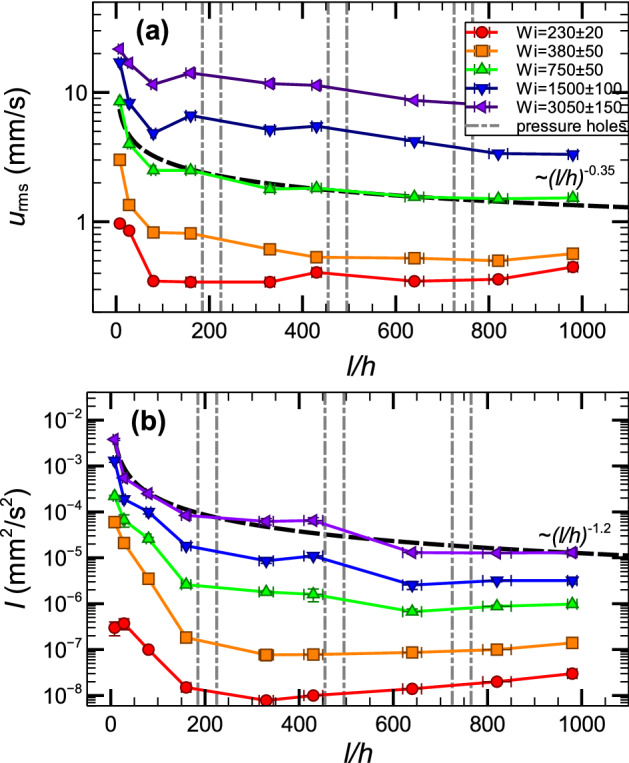
Decay of rms of velocity fluctuations $u_{rms}$ and elastic waves intensity *I* along the channel length for different *Wi*. Dependence of $u_{rms}$ (**a**) and *I* (**b**) on *l*/*h* at 5 values of *Wi*, presented in different colors, in three flow regimes: transition, ET, and DR. The $u_{rms}$ and *I* decays with *l*/*h* are algebraically fitted with the exponents of − 0.35 and − 1.2, respectively, as examples.

Over the whole channel length, both rms of stream-wise velocity fluctuations $u_{rms}$ and elastic wave intensity *I* decay downstream for five values of *Wi* in three flow regimes up to *l*/*h*=980, as shown in Fig. 6. The normalized rms, $u_{rms}/U_{mean}$, generated mostly by perturbations at the non-smoothed channel inlet and reached $u_{rms}/U_{mean}\approx 17\%$ at $l/h=0$, decay downstream down to $3-5\%$ at the outlet. One can also note a slight growth of both $u_{rms}$ and *I* near cavity locations indicated by the six dashed lines. The attenuation of $u_{rms}$ and *I* have fitted algebraically with the exponents of − 0.35 and − 1.2, respectively. The observed algebraic decay of *I* is slower than expected exponential attenuation due to viscous dissipation, possibly, as the result of perturbations generated by the six cavities along the channel length, which inject additional energy into both the flow and elastic waves. Finally, we found streaks at all *Wi* values, as shown in Fig. 6, downstream to $l/h=635$, suggesting that streaks may exist over the entire channel length.

Similar results are obtained in a straight channel with the smoothed inlet and very weak perturbations, generated by a single tiny cavity in a top plate at the middle of the channel, reported recently^27^. There the stream-wise streaks, $u_{rms}/U_{mean}$, and elastic waves are observed downstream the cavity up to $l/h\approx 210$. This observation range of the coherent structures and elastic waves is surprisingly comparable with $l/h\approx 200$ found in a similar straight channel with prearranged, strong perturbations at the inlet due to the obstacle array, and further downstream until the entire channel length only the stream-wise velocity fluctuations are detected in both cases^21,27^.

## Discussion

Now we return to the basic problems posed in the introduction. (i) We show that strong prearranged perturbations are not a necessary condition for the existence of the non-modal elastic instability in viscoelastic channel flows at $Wi\gg 1$ and $Re\ll 1$. Above the instability onset, the scaling behaviors of the friction factor, normalized velocity and pressure fluctuations with ($Wi/Wi_c-1$) in Fig. 2a,b with the exponents close to those found in Refs.^21,27^, and in particular, chaotic stream-wise velocity power spectra in Fig. 3a distinguish the instability as a non-normal mode. Moreover, the plots presented in Figs. 2 and 3 reveal three flow regimes above the instability onset: transition, ET, and DR, and all of them exhibit stream-wise streaks, similar to those observed in Refs.^23,27^. (ii) The velocity of stream-wise-propagating elastic waves has the same scaling exponent with $Wi-Wi_c$, as in Refs.^21,26,27^, suggesting universal scaling relation. (iii) The remarkable finding is uncovering the streaks and elastic waves in three flow regimes over the entire channel length up to *l*/*h*=980, in sharp contrast to the case of the strong prearranged perturbations at the inlet^21,23^, where the CSs and elastic waves are detected only in the restricted range of the channel from $l/h=36$ up to $l/h\approx 200$.

One of the central results, obtained in viscoelastic inertialess channel flows with various finite amplitude perturbations and reported here and in Refs.^21,27^, is the characterization of the supercritical non-modal instability directly to sustained chaotic flows observed throughout the full channel length. The latter validates that the instability is absolute without a prior convective instability, in contrast to all Newtonian parallel shear flows, where the subcritical instability caused by finite-size perturbations to a transition phase in an extensive range of *Re* from transient turbulence up to the commencement of sustained turbulence^8,22,28,29^. In light of these two reasons, the direct instability to sustained chaotic flows differs significantly from the extensive transition region in Newtonian parallel shear flows. In the transient turbulent region above the subcritical instability, the flow appears as localized turbulent spots (puffs) embedded into a laminar flow^8,22^. At higher *Re*, first convective and then further absolute instabilities are observed both theoretically and experimentally^22,28,29^. In the convective instability region, a pulse perturbation propagates and spatially spreads as a turbulent spot downstream without spreading upstream and temporally decays locally. At further *Re* increase, in the absolute instability region, the pulse spreads upstream and downstream leading eventually to sustained turbulence^8,22,28,29^.

Thus, even though Newtonian and viscoelastic shear flows are both open, the latter exhibits the direct transition to sustained chaotic flows via exclusively absolute instability. Our explanation for the existence of only the absolute instability in viscoelastic inertialess channel flows, described by the set of Stokes and elastic stress equations at $Wi\gg 1$ and $Re\ll 1$, versus the Newtonian NSE at $Re\gg 1$, is the crucial difference between them^4^. NSE at $Re\gg 1$ has just one nonlinear advection term, whereas the elastic stress nonlinear equation at $Wi_c$ contains three nonlinear terms^4^: One term describes an advection of the stress perturbations, while two other terms describe polymer stretching by velocity gradients resulting in an increase of the elastic stress over the whole channel^4^. It means that two nonlinear terms absent in NSE cause the amplification of the elastic stress field throughout the whole channel despite its advection. Due to linear feedback introduced by an additional term of the elastic stress gradient in the Stokes equation, the velocity field is affected by the growing elastic stress leading to absolute instability^4^.

In conclusion, we provide experimental evidence validating the similarity of inertialess viscoelastic channel flows with strong and weak perturbations at the inlet at $Wi\gg 1$ and $Re\ll 1$. The discovered resemblance in a direct transition to sustained chaotic flows, characterized by continuous velocity power spectra and about the same scaling exponent values of the flow parameters, distinguishable from the normal mode scaling, clearly confirms the non-normal mode nature of the transition. Along with the discovery of streaks at $Wi>Wi_c$ in three flow regimes and the universal scaling exponent of the elastic wave propagation velocity with $Wi-Wi_c$, it supports universality in the scaling characteristics and structures of planar channel flows of viscoelastic fluids, regardless of the perturbation intensity. Moreover, the absence of convective instability in inertialess viscoelastic channel flows is the most surprising discovery.

## Methods

### Experimental setup, measurement techniques, and preparation of polymer solution

The experiments are conducted in a straight channel of 500(*L*) $\times$ 3.5(*w*) $\times$ 0.5(*h*) mm$^3$ dimensions, shown in Fig. 1. The fluid is driven by N$_2$ gas at pressure up to 100 psi. The exiting fluid is weighed instantaneously, *m*(*t*), by a PC-interfaced balance (BPS-1000-C2-V2, MRC) to measure the time-averaged fluid discharge rate $Q=\langle \Delta m/\Delta t\rangle$ to get the mean velocity $U_{mean}=Q/\rho w h$. Then $Wi=\lambda U_{mean}/ h$ and $Re=\rho U_{mean} h/\eta$ vary in the ranges (30, 6000) and (0.005, 0.9), respectively. High resolution ($0.1\%$ of full scale) differential pressure sensors (HSC series, Honeywell) of different ranges: 5, 30, and 60 psi, are used to measure the pressure drop $\Delta P$ and fluctuations.

### Preparation and characterization of polymer solution

As a working fluid, a dilute polymer solution of high-molecule-weight Polyacrylamide (Polysciences, $M_w=18 MDa$) at concentration $c=80$ ppm ($c/c*\approx 0.4$ with the overlap polymer concentration $c*\approx 200$ ppm^30^) is prepared using a water-sucrose solvent with $64\%$ weight fraction. The solution properties are the solution density $\rho =1320$ kg/m$^3$, the solvent and solution viscosity $\eta _s=0.13$ Pa$\cdot$s and $\eta = \eta _s+\eta _p=0.17$ Pa$\cdot$s, respectively, $\eta _p/(\eta _s+\eta _p)\approx 0.3$, where $\eta _p$ is the polymer contribution into the solution viscosity, and the longest polymer relaxation time $\lambda =13$ s using the stress relaxation method^30^.

### Imaging system and PIV measurements

We conduct velocity field measurements at various distances *l*/*h* downstream from the inlet, using the particle image velocimetry (PIV) method. Small latex fluorescent particles (3.2$\mu$m) tracers of $\sim$ 0.67% w/w concentration (Thermo Scientific) illuminated by a laser sheet of $\approx 100 \mu$m thickness over the middle channel plane. A high-speed camera (Mini WX100 FASTCAM, Photron) with high spatial resolution (up to 2048$\times$2048 px$^2$) using from 500 up to 15000 fps is used to capture pairs of tracer images. The OpenPIV software^31^ is employed to analyze *u*(*x*, *z*, *t*) and *w*(*x*, *z*, *t*) in 2D *x*-*z* plane to record data for $\sim {\mathcal {O}}(15)$ minutes, or $\sim {\mathcal {O}}(50\lambda )$, for each *Wi* to obtain sufficient statistics.

## Data Availability

The data that support the findings of this study are available from the corresponding authors upon reasonable request.
